# Effect of Vitamin A Supplementation on Growth Performance, Serum Biochemical Parameters, Intestinal Immunity Response and Gut Microbiota in American Mink (*Neovison vison*)

**DOI:** 10.3390/ani11061577

**Published:** 2021-05-28

**Authors:** Weixiao Nan, Huazhe Si, Qianlong Yang, Hongpeng Shi, Tietao Zhang, Qiumei Shi, Guangyu Li, Haihua Zhang, Hanlu Liu

**Affiliations:** 1 Department of Special Animal Nutrition and Feed, Institute of Special Animal and Plant Sciences, Chinese Academy of Agricultural Sciences, Changchun 130112, China; nanwx2015@126.com (W.N.); sihuazhe1989@163.com (H.S.); qianlongy@126.com (Q.Y.); shihongpeng1994@126.com (H.S.); pheilty@163.com (T.Z.); tcslgy@126.com (G.L.); 2 College of Animal Science and Technology, Jilin Agricultural University, Changchun 130118, China; 3 College of Animal Science and Technology, Hebei Normal University of Science and Technology, Qinhuangdao 066004, China; shiqiumei@126.com

**Keywords:** vitamin A, mink, growth performance, *IL-22*, villus height, *Akkermansia*

## Abstract

**Simple Summary:**

Vitamin A is critical throughout life, but utilization of vitamin A often results in local and systemic toxicity. This study investigated the effect of vitamin A supplementation on mink growth and health. The results show that vitamin A deficiency decreased the ADG, villus height, villus height/crypt depth ratio and mRNA expression levels of *IL-22*, *Occludin* and *ZO-1*. Vitamin A supplementation increased the diversity of jejunum bacteria, decreased the ratio of *Firmicutes* to *Bacteroidetes* and increased the relative abundance of *Akkermansia* and *Lachnospiraceae NK4A136 group*.

**Abstract:**

This experiment investigated the effect of vitamin A supplementation on growth, serum biochemical parameters, jejunum morphology and the microbial community in male growing-furring mink. Thirty healthy male mink were randomly assigned to three treatment groups, with 10 mink per group. Each mink was housed in an individual cage. The mink in the three groups were fed diets supplemented with vitamin A acetate at dosages of 0 (CON), 20,000 (LVitA) and 1,280,000 IU/kg (HVitA) of basal diet. A 7-day pretest period preceded a formal test period of 45 days. The results show that 20,000 IU/kg vitamin A increased the ADG, serum T-AOC and GSH-Px activities, villus height and villus height/crypt depth ratio (*p* < 0.05). The mRNA expression levels of *IL-22*, *Occludin* and *ZO-1* in the jejunum of mink were significantly higher in the LVitA group than those in the CON and HVitA groups (*p* < 0.05). Vitamin A supplementation increased the diversity of jejunum bacteria, decreased the ratio of *Firmicutes* to *Bacteroidetes* and increased the relative abundance of *Akkermansia*, *uncultured bacterium f Muribaculaceae*, *Allobaculum*, *Lachnospiraceae NK4A136 group*, *Rummeliibacillus* and *Parasutterella*. The comparison of potential functions also showed enrichment of glycan biosynthesis and metabolism, transport and catabolism pathways in the vitamin A supplementation groups compared with the CON group. In conclusion, these results indicate that dietary vitamin A supplementation could mediate host growth by improving intestinal development, immunity and the relative abundance of the intestinal microbiota.

## 1. Introduction

Vitamin A and its metabolites regulate diverse processes, including reproduction, embryogenesis, vision, growth, cellular differentiation and proliferation in mammals [1,2,3]. Vitamin A also plays an important role in intestinal immunity and epithelial integrity, and promotes healthy colonization of the intestinal mucosa with commensal bacteria [4]. Clearly, vitamin A is critical throughout life, together with natural derivatives and synthetic analogs [2]. At present, vitamin A deficiency is a worldwide public health problem that can cause micronutrient malnutrition, slow growth impairing innate immunity and adverse health consequences for people and animals [5,6]. Deficiency in vitamin A and its metabolites can cause abnormal morphological development [7,8]. However, excessive utilization of vitamin A often results in local and systemic toxicity [9]. Thus, understanding how vitamin A levels are regulated has important practical implications.

Vitamin A has three active forms (retinal, retinol and retinoic acid), and vitamin A metabolism can be classified into three major processes—intestinal uptake (especially small intestine), hepatic storage and lymphatic and blood transport to supply the body’s physiological needs [10]. However, mammals cannot synthesize vitamin A in the body and can only obtain vitamin A from the diet. The two main forms of vitamin A in the diet are retinyl/retinol esters (animal products), and provitamin A carotenoids (plants) [11,12]. Notably, the cleavage enzyme (beta-carotene-15,15′-monooxygenase) has been found in many vertebrates but is not present in mink, so mink cannot utilize carotene as a source of vitamin A [13,14]. Therefore, the nutritional requirement of vitamin A for mink must be obtained from a diet containing preformed retinol.

The American mink (*Neovison vison*), an obligate carnivore, has a short and simple gastrointestinal tract [15,16], and little is known regarding the structural and functional adaptation of the gastrointestinal tract. Gut health is influenced by the maintenance of the delicate balance between the host, intestinal microbiota, intestinal barrier and dietary compounds [17]. The gut microbiota performs numerous beneficial functions for the host, such as harvesting energy, regulating immunity and aiding cellular maturation [18,19]. However, the gut microbial community is influenced by various parameters, such as the host diet composition and type, lifestyle, antibiotics and other drugs, while the genetics and immune status of the host also shape the microbiota composition, with various consequences for host physiology [18,20,21,22]. The gut microbial community composition of carnivores in general appears to be distinct from that of omnivores and herbivores [23]. Previous research found that vitamin A could regulate the interactions between eukaryotic host cells and symbiotic microbes, as well as the complexity of the microbiome, and the microbiome regulates vitamin A metabolism in the host [24,25]. In our previous study, supplementation with vitamin A at 0 and 1,280,000 IU/kg had a negative impact on the growth performance and digestion of dry matter, crude protein and ether extract compared with 20,000 IU/kg in growing-furring mink [26]. Therefore, we hypothesized that vitamin A can affect the growth of mink by modulating intestinal development, immune function and the intestinal microbiome.

Thus, the present study aims to examine the effects of vitamin A supplementation (1) on growth performance and serum biochemical parameters and (2) on jejunum morphology and gut microbiota of American mink during the growing-furring period.

## 2. Materials and Methods

### 2.1. Animals, Experimental Design and Diets

Thirty 15-wk-old healthy male mink (BW = 1.98 ± 0.04 kg), approved by the Institute of Special Animal and Plant Sciences, Chinese Academy of Agricultural Science (CAAS), were randomly assigned to three treatment groups, with 10 animals per group. The mink were housed in open-sided sheds in individual mink growing cages (60 cm long × 40 cm wide × 50 cm high) with attached nest boxes (30 cm long × 40 cm wide × 30 cm high). The mink were fed twice each day at 8:00 and 15:00 and had free access to water. After 7 days of adaptation, the experiment lasted for 45 days from late September to pelting in late November.

The diets were formulated based on the Management Guide of the National Research Council (NRC, 1982) [13] with no supplemental vitamin A. The ingredients used were extruded corn, soybean meal, meat meal, meat and bone meal, corn gluten meal, fish meal and soybean oil (Table 1). The mink in each group were fed a basal diet with 0 (CON), 20,000 (LVitA) and 1,280,000 IU/kg (HVitA) vitamin A acetate (DSM Vitamin Co., Ltd. Shanghai, China).

### 2.2. Experimental Procedure and Sample Collection

The animals were weighed biweekly before morning feeding, and the final body weight of mink was used to determine average daily gain (ADG). After 45 days, five mink from each group were selected randomly, and euthanized by electrocution according to the Welfare of Animals Kept for Fur Production requirements [27]. Body length was measured from the base of the tail to the tip of the nose. Immediately after death, blood samples (6 mL) were collected from the heart of each mink from the three groups into coagulation tubes and centrifuged at 3000× *g* for 15 min to obtain the serum. All serum samples were stored at −20 °C for later analysis. Jejunum tissue was removed immediately and placed in 10% formalin as a fixative for histology. The content and mucosa of the jejunum were collected in a sterile tube, transferred into liquid nitrogen and then stored at −80 °C for further analysis.

### 2.3. Chemical Analysis

Diet samples were dried at 65 °C in a forced air oven to reach a constant weight and kept for further analysis. All chemical analyses were conducted in duplicate. Diet samples were analyzed for dry matter (AOAC, 2000 method 930.15), crude protein (AOAC, 2000 method 984.13), ether extract (AOAC, 2000 method 920.39) and ash (AOAC, 2000 method 942.05). The concentration of vitamin A from feedstuffs was analyzed by ultra-performance liquid chromatography according to a published procedure [26].

### 2.4. Measurement of Serum Samples

The concentrations of immunoglobulins (IgA and IgM), complement levels (C3 and C4), total antioxidant capacity (T-AOC), glutathione peroxidase (GSH-Px) and malondialdehyde (MDA) activities in serum were measured by colorimetric methods using a Microplate Spectrophotometer (Epoch 2, BioTek Instruments Inc, Winooski, VT, USA) following the manufacturer’s instructions, provided by Nanjing Jiancheng Biochemical Corporation (Nanjing, Jiangsu, China).

### 2.5. Measurement of Jejunum Morphology

Jejunum samples were dehydrated and paraffin-embedded (Thermo A81010100 Issue2). The paraffin-embedded blocks were sectioned at 7 μm by a microtome (Thermo HM340E). Jejunum sections were stained with hematoxylin and eosin (H&E) to evaluate their general histological structure. A light microscope (BX51, Olympus Co., Tokyo, Japan) was used for bright field imaging. The jejunum villus height (Vh), crypt depth (Cd) and intestinal wall thickness were determined using an image analysis program (Image-Pro Plus 5.1, Rockville, MD, USA), and the Vh/Cd ratio was calculated.

### 2.6. Total RNA Extraction and Quantitative Real-Time Polymerase Chain Reaction (qPCR)

Total RNA of the jejunum mucosa samples of mink was extracted using TRIzol reagent (Takara Biotechnology Co. Ltd., Dalian, China), according to the manufacturer’s protocol. First-strand complementary DNA was then synthesized using the PrimeScript™ RT reagent Kit with gDNA Eraser (Perfect Real Time) (TaKaRa, Dalian, China) following the manufacturer’s instructions. Real-time PCR was performed on StepOnePlus Real-Time PCR Systems (Applied Biosystems, Foster City, CA, USA) to quantify *IL-22*, *zonula occludens-1* (*ZO-1*) and *Occludin* mRNA expression with TB Green^®^ Premix Ex Taq™ II (Tli RNaseH Plus) (TaKaRa, Dalian, China). Relative expression levels were calculated using the 2^−ΔΔCT^ method [28] and normalized to *GAPDH*, and all reactions were run in triplicate. The primers were synthesized by Sangon Biotech (Changchun, China). Primers were as follows: *IL-22* forward: TGCCTCCTCATTGCCCTGTG, reverse: AGCATGAAGGTGCGGTTGGT; *Occludin* forward: TGACCTCGCCCGTGGATGACTT, reverse: TTGGACCTGCCTGCTCTGCCTT; *ZO-1* forward: CTCCTCTAATACCTGCGTCTCA, reverse: TTCATCCTTCTTGCTCTCCAATG; and *GAPDH* forward: GAAGGTGGTGGCGGTGAATGAT, reverse: TCTTGGGTGGCAAGGGTGGA.

### 2.7. DNA Extraction, Amplification, Sequencing and Bioinformatics Analysis

The microbial genomic DNA in the jejunum was extracted according to the manufacturer’s instructions using a Fast DNA Spin Kit (MP, Valencia, CA, USA). The primers 338F (5′- ACTCCTACGGGAGGCAGCA-3′) and 806R (5′-GGACTACHVGGGTWTCTAAT-3′) were used to amplify the V3–V4 region of the bacterial 16S rRNA gene. The resultant amplicons were purified using a QIAquick PCR Purification Kit (QIAGEN, Valencia, CA, USA), and then sequenced on an Illumina NovaSeq 6000 platform to produce 250-bp paired-end reads.

The paired-end sequences were first assembled into contigs using FLASH v1.2.7 [29] and then used for quantitative insights into microbial ecology (QIIME v1.9.1) [30]. The sequences were clustered into operational taxonomic units (OTUs) using UPARSE at 97% sequence similarity. Potential chimeric sequences were identified and removed using UCHIME [31]. The representative sequences of each OTU were assigned against the SILVA database (v138) using the RDP classifier with a 0.80 confidence threshold [32]. Alpha diversity indices, including the Chao 1, ACE, Shannon and Simpson indices, were calculated using QIIME v1.9.1 [30]. Principal coordinate analysis (PCoA) based on four distances was used to reveal the differences in the bacterial communities among the three groups [33]. Analysis of similarities (ANOSIM) was performed to indicate group similarity, where 0 = indistinguishable and 1 = dissimilar [34]. Adonis was employed to describe the strengths and significance of the differences among the microbial communities. For the ANOSIM and Adonis analyses, the *p*-values were determined based on 999 permutations. The sequences from the present study have been deposited in the SRA database. The reconstruction of unobserved states (PICRUSt) was applied to predict functional profiles of gut microbiota resulting from reference-based OTU picking against the Greengenes database [35]. The predicted genes were then summarized according to Kyoto Encyclopedia of Genes and Genomes (KEGG) pathways.

### 2.8. Statistical Analysis

All graphs were generated using GraphPad Prism version 6.01 (GraphPad Software, Inc., San Diego, CA, USA) [36]. Statistical analyses were performed using SPSS Statistical 24.0 (IBM, New York, NY, USA) [37] with diet as the main effect. The results of the statistical analysis are presented as the means with their standard errors, and the error bars are standard deviations. The data were analyzed using one-way analysis of variance (ANOVA). Duncan’s tests were used to detect statistical significance between treatment groups (CON, LVitA and HVitA groups). The p-values were corrected using the false discovery rate of the Benjamini–Hochberg method. A significant difference between treatments was declared when *p* < 0.05.

## 3. Results

### 3.1. Growth Performance and Serum Biochemical Constituents

**Figure 1:**
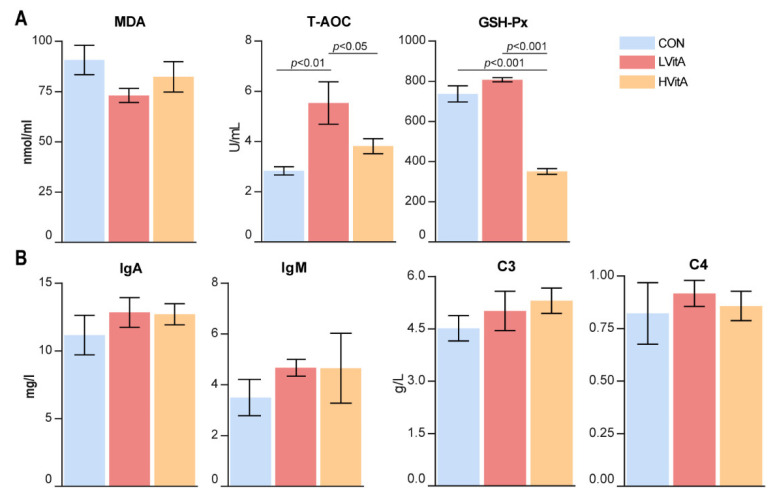
Effect of vitamin A on serum antioxidant capacity (**A**) and immunological indices (**B**) of growing-furring male mink. CON group, vitamin A with 0 IU/kg of diet; LVitA group, vitamin A with 20,000 IU/kg of diet; HVitA group, vitamin A with 1,280,000 IU/kg of diet. MDA, malondialdehyde; T-AOC, total antioxidant capacity; GSH-Px, glutathione peroxidase; IgA, immunoglobulin A; IgM, immunoglobulin M; C3, complement C3; C4, complement C4.

**Figure 2:**
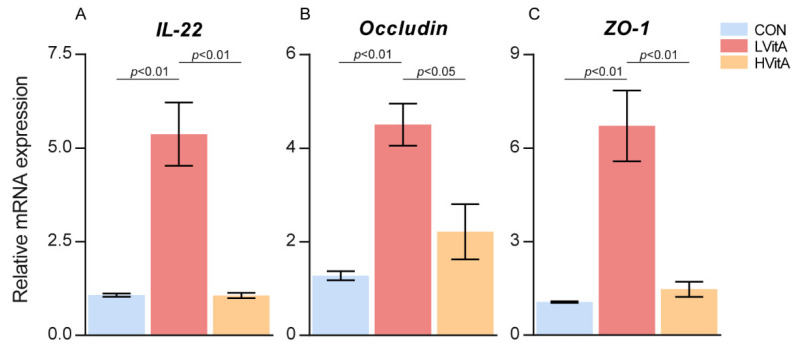
Effect of vitamin A on the mRNA levels of *IL-22* (**A**), *Occludin* (**B**) and *ZO-1* (**C**) in the mink jejunum. CON group, vitamin A with 0 IU/kg of diet; LVitA group, vitamin A with 20,000 IU/kg of diet; HVitA group, vitamin A with 1,280,000 IU/kg of diet.

The results show that the ADG was significantly higher in the LVitA group than that in the CON group (*p* < 0.05, Table 2). There were no significant differences among the three groups in final body weight and body length (*p* > 0.05). The activity of T-AOC in the LVitA and HVitA groups was significantly higher than that in the CON group, whereas the GSH-Px activity was significantly decreased in the HVitA group compared with the CON and LVitA groups (*p* < 0.05, Figure 1A). There were no significant differences among the three groups in MDA activity (*p* > 0.05, Figure 2A) and the concentrations of IgA, IgM, C3 and C4 (*p* > 0.05, Figure 1B) in serum.

### 3.2. Morphometric Analysis and mRNA Expression of IL-22, Occludin and ZO-1 in the Jejunum

As shown in Table 3, the villus height was significantly higher (*p* < 0.01) in the LVitA group than that in the CON and HVitA groups. The villus height:crypt depth ratio was significantly lower in the CON group than that in the CON and HVitA groups (*p* < 0.05). There was no significant difference among the three groups in crypt depth and intestinal wall thickness (*p* > 0.05). The jejunal mucosa mRNA expression levels of *IL-22*, *Occludin* and *ZO-1* were significantly higher in the LVitA group than those in the CON and HVitA groups (*p* < 0.05, Figure 2).

### 3.3. Summary of High-Throughput Sequencing and Alpha Diversity

**Figure 3:**
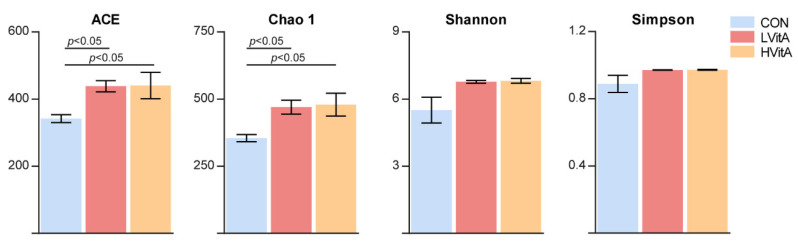
Comparisons of the alpha diversity indices of the mink gut microbiota among the three groups. CON group, vitamin A with 0 IU/kg of diet; LVitA group, vitamin A with 20,000 IU/kg of diet; HVitA group, vitamin A with 1,280,000 IU/kg of diet.

The present study obtained a total of 1,170,905 16S rRNA gene sequences from three groups, with a range of 72,190 to 79,412 sequences for each sample. We subsampled the sequences in each sample to 72,190 to decrease the effect of sequencing depth. A total of 920 OTUs were identified at 97% sequence similarity and represented 20 phyla and 340 genera in the three groups. Good’s coverage, ranging from 0.993 to 0.999, demonstrated an adequate sequencing depth for all samples. The number of OTUs was significantly higher in the LVitA and HVitA groups than that in the CON group (*p* < 0.05, CON = 293.60 ± 39.07, LVitA = 372.60 ± 16.43 and LVitA = 371.80 ± 42.32, respectively). As shown in Figure 3, the ACE and Chao1 indices in the CON group were significantly lower than those in the LVitA and HVitA groups (*p* < 0.05). There was no difference among the three groups in the Shannon and Simpson index values (*p* > 0.05).

### 3.4. Dietary Vitamin A Altered the Composition and Function of the Gut Microbiota in Mink

**Figure 4:**
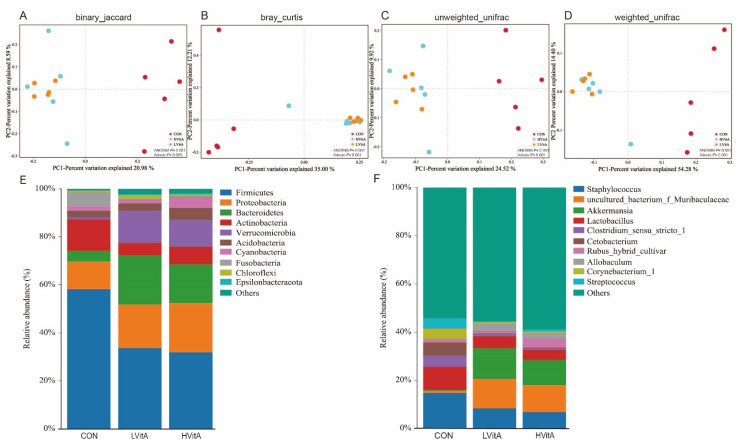
Composition and comparisons of the mink gut microbiota among the three groups. PCoA revealing the separation of the gut microbiota in the three groups based on the binary Jaccard distance (**A**), Bray–Curtis distance (**B**), unweighted UniFrac distance (**C**) and weighted UniFrac distance (**D**). Microbial compositions in the gut of mink from the CON, LVitA and HVitA groups at the phylum (**E**) and genus (**F**) levels. CON group, vitamin A with 0 IU/kg of diet; LVitA group, vitamin A with 20,000 IU/kg of diet; HVitA group, vitamin A with 1,280,000 IU/kg of diet.

PCoA was applied to examine differences in taxonomic community composition and structure in the jejunum of mink. The PCoA based on the binary Jaccard distance (Figure 4A, ANOSIM: *p* = 0.001; Adonis: *p* = 0.005), Bray–Curtis distance (Figure 4B, ANOSIM: *p* = 0.001; Adonis: *p* = 0.001), unweighted UniFrac distance (Figure 4C, ANOSIM: *p* = 0.001; Adonis: *p* = 0.001) and weighted UniFrac distance (Figure 4D, ANOSIM: *p* = 0.001; Adonis: *p* = 0.001) showed that the CON group was clearly separated from the LVitA and HVitA groups.

At the phylum level, *Firmicutes* was the dominant bacteria in all three groups (58.2, 33.6 and 32.2%, respectively), while vitamin A supplementation affected the following abundant phyla in the treatment groups compared with the CON group (Figure 4E): (Group CON) *Actinobacteria* (12.9%), *Proteobacteria* (11.4%), *Fusobacteria* (5.5%) and *Bacteroidetes* (4.6%); (Group LVitA) *Bacteroidetes* (20.4%), *Proteobacteria* (18.4%), *Verrucomicrobia* (13.2%) and *Actinobacteria* (5.3%); and (Group HVitA) *Proteobacteria* (20.7%), *Bacteroidetes* (15.7%), *Verrucomicrobia* (11.1%) and *Actinobacteria* (7.2%). The ratio of *Firmicutes* to *Bacteroidetes* in the vitamin A supplementation groups (LVitA = 1.66, HVitA = 1.97, respectively) significantly decreased compared with that in the CON group (12.97). Moreover, vitamin A supplementation also significantly affected the genera in mink (*p* < 0.05, Figure 4F). For instance, *Staphylococcus* (14.7%), *Lactobacillus* (9.5%), *Cetobacterium* (5.4%), *Clostridium* sensu stricto *1* (4.8%) and *Corynebacterium 1* (4.4%) were the five most abundant genera in the CON group, while the genera *Akkermansia* (LVitA = 12.7%, HVitA = 10.43%, respectively), *uncultured bacterium f Muribaculaceae* (LVitA = 12.3%, HVitA = 11.2%, respectively), *Staphylococcus* (LVitA = 8.3%, HVitA = 6.7%, respectively) and *Lactobacillus* (LVitA = 5.08%, HVitA = 4.31%, respectively) were the most abundant in both the LVitA and HVitA groups, followed by *Allobaculum* (3.1%) in the LVitA group and *Rubus hybrid cultivar* (3.8%) in the HVitA group.

**Figure 5:**
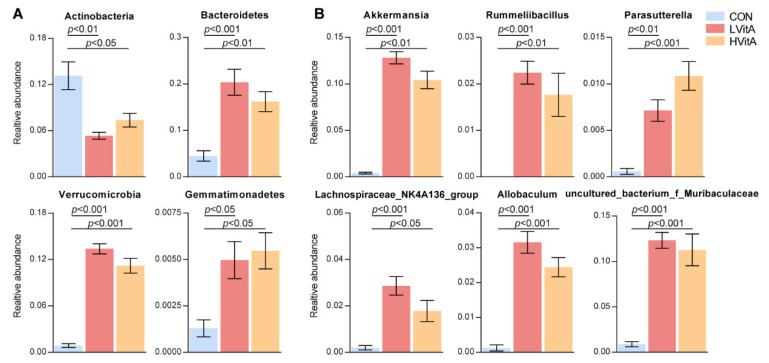
Histogram of ANOVA among the CON, LVitA and HVitA groups at the phylum (**A**) and genus (**B**) levels. CON group, vitamin A with 0 IU/kg of diet; LVitA group, vitamin A with 20,000 IU/kg of diet; HVitA group, vitamin A with 1,280,000 IU/kg of diet.

Furthermore, we also compared the bacterial taxa among the three groups. The relative abundances of *Bacteroidetes* (CON = 4.48%, LVitA = 20.34%, HVitA = 16.20%, respectively), *Gemmatimonadetes* (CON = 0.13%, LVitA = 0.50%, HVitA = 0.55%, respectively) and *Verrucomicrobia* (CON = 0.87%, LVitA = 13.38%, HVitA = 11.20%, respectively) were significantly increased (*p* < 0.05), while the relative abundance of *Actinobacteria* (CON = 13.15%, LVitA = 5.33%, HVitA = 7.36%, respectively) was significantly decreased in the vitamin A supplementation groups compared with the CON group (Figure 5A). At the genus level, six genera were significantly increased (*p* < 0.05) in the LVitA and HVitA groups compared with the CON group, namely *Akkermansia* (CON = 0.41%, LVitA = 12.81%, HVitA = 10.41%, respectively), *uncultured bacterium f Muribaculaceae* (CON = 0.88%, LVitA = 12.33%, HVitA = 11.27%, respectively), *Allobaculum* (CON = 0.13%, LVitA = 3.16%, HVitA = 2.44%, respectively), *Lachnospiraceae NK4A136 group* (CON = 0.20%, LVitA = 2.87%, HVitA = 1.78%, respectively), *Rummeliibacillus* (CON = 0, LVitA = 2.24%, HVitA = 1.77%, respectively) and *Parasutterella* (CON = 0.06%, LVitA = 0.71%, HVitA = 1.09%, respectively) (Figure 5B).

### 3.5. Comparison of the Potential Functions in the Jejunal Microbiota among the Three Groups

**Figure 6:**
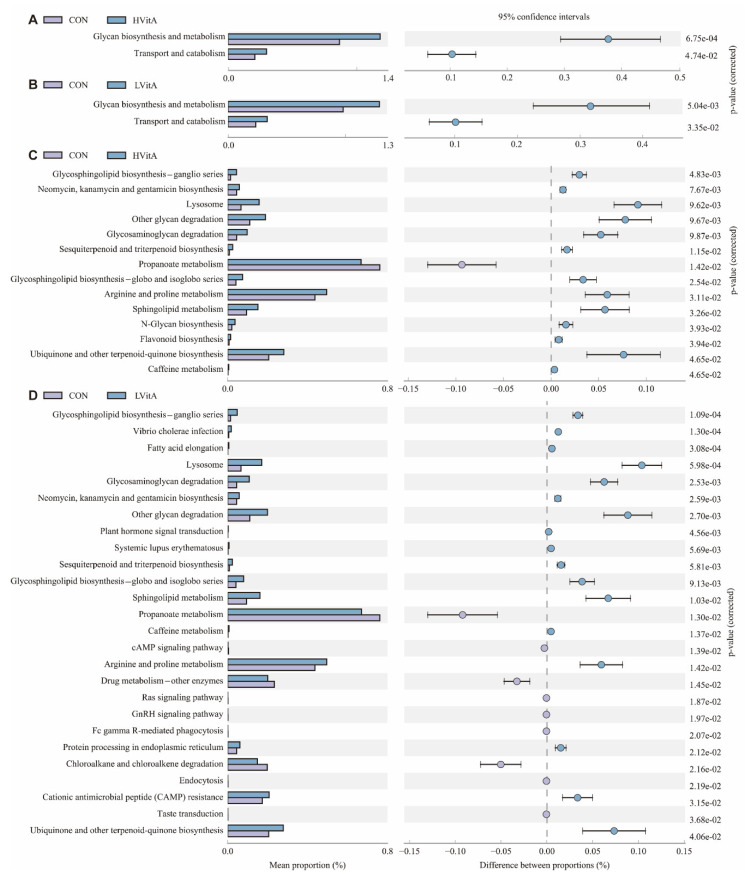
Comparison of KEGG pathways predicted by PICRUSt according to diet. The differential analysis diagram of KEGG metabolic pathways between the CON and HVitA groups at levels 2 (**A**) and 3 (**C**) and the differential analysis diagram of KEGG metabolic pathways between the CON and LVitA groups at levels 2 (**B**) and 3 (**D**). CON group, vitamin A with 0 IU/kg of diet; LVitA group, vitamin A with 20,000 IU/kg of diet; HVitA group, vitamin A with 1,280,000 IU/kg of diet.

KEGG annotation by PICRUSt was applied to analyze the functional differences among the three groups. The KEGG level 2 functional pathways showed that glycan biosynthesis and metabolism, transport and catabolism pathways were significantly more abundant in the vitamin A supplementation groups than those in the CON group (Figure 6A,B). A total of 14 KEGG metabolic pathways (level 3) were enriched in the HVitA group compared with the CON group (Figure 6C). Among the pathways belonging to the top 10 dominant KEGG pathways, the relative abundance of glycosphingolipid biosynthesis—ganglio series, neomycin, kanamycin and gentamicin biosynthesis, lysosome, other glycan degradation, glycosaminoglycan degradation, sesquiterpenoid and triterpenoid biosynthesis, glycosphingolipid biosynthesis—globo and isoglobo series, arginine and proline metabolism and sphingolipid metabolism were significantly higher, while the relative abundance of propanoate metabolism was lower in the HVitA group than in those in the CON group. A total of 26 KEGG metabolic pathways (level 3) were enriched in the LVitA group compared with the CON group (Figure 6D). Among the pathways belonging to the top 10 dominant KEGG pathways, the relative abundance of glycosphingolipid biosynthesis—ganglio series, vibrio cholerae infection, fatty acid elongation, lysosome, glycosaminoglycan degradation, neomycin, kanamycin and gentamicin biosynthesis, other glycan degradation, plant hormone signal transduction, systemic lupus erythematosus, sesquiterpenoid and triterpenoid biosynthesis in the LVitA group were significantly higher than those in the CON group.

## 4. Discussion

The ADG in the CON group was significantly lower than that in the LVitA and HVitA groups. In the present study, the content of vitamin A in the CON group was lower than the NRC requirement (100–400 IU of vitamin A per kilogram of body weight daily), which may cause vitamin A deficiency in mink [13]. Vitamin A deficiency affects nutrient metabolism, intestinal immunity and epithelial integrity and modulates microbiome development in rats and humans [38,39,40]. Similarly, Tian et al. [41] also found that rats fed a vitamin A-deficient diet had decreased body weight. This evidence demonstrated that vitamin A deficiency caused a negative effect on growth performance in mammals. In this experiment, the results show that 20,000 IU/kg vitamin A supplementation had no effect on the body length of mink. A previous study observed that the epiphyses of the long bones of the mink were close to approximately 16 weeks of age; thus, actual body length cannot be increased beyond this point, although body weight may be enhanced [42]. In this experiment, thirty 15-wk-old mink had an insignificant initial weight difference, which might be the reason why the body length was not significant according to the ANOVA analyzed.

The results show that vitamin A supplementation affected the antioxidant activities in American mink. The T-AOC activity in the LVitA group was greater than that in the CON and HVitA groups. Consistent with our finding, Liang et al. [43] found that vitamin A supplementation significantly increased the activity of T-AOC in goslings. T-AOC is the total ability of various antioxidants to scavenge oxygen free radicals in enzymatic and non-enzymatic systems [44,45], and acts as an effective quencher and scavenger of lipid peroxidation free radicals, hydroxyl radicals and other free radicals [46]. We also observed that the activity of GSH-Px was significantly lower in the HVitA group than that in the CON and LVitA groups. GSH-Px has the ability to prevent cellular damage from oxidative stress by scavenging free radicals in animals [47]. Based on GSH-Px activity in the present study, although high doses of vitamin A supplementation showed no negative effect on growth performance, it still caused toxicity in mink [48]. Excess vitamin A supplementation resulted in abnormalities in hematologic and biochemical indices [49]. Combining the growth performance and antioxidant index results of the present study seems to suggest that mink is a carnivore that may be most tolerant to high dietary vitamin A [50].

In the present study, we observed that 20,000 IU/kg vitamin A supplementation significantly increased the Vh and Vh/Cd ratio. As indicators of intestinal health or morphology, Vh, Cd and the Vh/Cd ratio are related to intestinal health [51]. The small intestine not only serves as the main site of liquid feed digestion but also plays a role in nutrition absorption. Increased Vh may improve nutrient absorption and performance [51,52]. The Vh/Cd ratio is an important value for evaluating the developmental state of the intestine [53]. However, our results also show that a deficiency (0 IU/kg) and high dietary vitamin A levels (1,280,000 IU/kg) decreased the Vh and Vh/Cd ratio. These results may be due to a deficiency of vitamin A causing intestinal epithelial damage [54], while a large dose of vitamin A exceeded the absorption and circulation capacity of the intestine in a single feeding episode [55].

Furthermore, the relative mRNA expression levels of immune- and tight junction-related genes in the jejunum mucosa were measured to explore intestinal health in mink. The results show that the relative expression of *IL-22* in the CON and HVitA groups was significantly lower than that in the LVitA group. Previous studies indicated that vitamin A plays an important role in the signaling of *IL-22* in the gut [56]. IL-22 is a protective cytokine that exerts a protective role in mucosal immunity and coordinates the antimicrobial response against gut bacteria [57,58]. Consistent with previous research [59,60], a 20,000 IU/kg vitamin A addition significantly increased the relative expression of *ZO-1* and *Occludin* in the jejunum. *Occludin* and *ZO-1* can seal the intercellular gaps between adjacent endothelial cells and form a selectively permeable barrier for circulating molecules [61]. A previous study indicated that vitamin A and its metabolites regulate the expression of tight junction proteins on intestinal epithelial cells for barrier function in the gut [24]. These results suggest that vitamin A has protective effects on intestinal epithelial barrier function. However, under the condition of vitamin A deficiency, mucosal immune responses are reduced in animals [62].

Establishing and maintaining beneficial interactions between the host and its associated microbiota are important factors affecting host health [19]. The metabolic activities of the gut microbiota result from extracting calories from ingested dietary substances, helping to store those calories in host adipose tissue for later use and providing energy and nutrients for microbial growth and proliferation [63]. Previous studies have demonstrated that microbiota alterations exert profound effects on host physiology and metabolism [64,65,66,67,68]. In the present study, there were significant differences in microbial community composition in the jejunal microflora of American mink between the CON and treatment groups, which is consistent with previous research on the cecal microbiota of mice [41]. Moreover, the treatment groups significantly increased the alpha diversity of the gut microbiota, including the Chao1 and ACE indices.

At the phylum level, the jejunum microbiomes of mink evaluated here were dominated by sequences representative of *Firmicutes*, *Proteobacteria*, *Bacteroidetes* and *Actinobacteria*, consistent with observations in the colon [69] and feces [15] of mink. The phyla *Firmicutes*, *Proteobacteria*, *Bacteroidetes* and *Actinobacteria* were also widely present in the gastrointestinal tract of other carnivore species, such as Eurasian otters, leopard cats, raccoon dogs, lion and silver fox [70,71,72,73]. Consistent with results in mice [41], we also found that the relative abundance of *Verrucomicrobia* and *Bacteroidetes* significantly increased in the treatment groups compared with the CON group. Moreover, the abundance of *Actinobacteria* in the LVitA and HVitA groups was lower than that in the CON group. Consistent with Xiao et al. [74], the percentage of *Actinobacteria* was highest in the vitamin A deficiency group. Moreover, the *Firmicutes*/*Bacteroidetes* ratio (F/B ratio) was also decreased in the vitamin A supplementation groups compared with the CON group, consistent with observations in the cecum of mice [41]. Previous studies have shown that the F/B ratio is directly related to dysbiosis, the disruption of immunological homeostasis and host energy metabolism [66,75,76,77].

At the genus level, our results show that the relative abundance of *Akkermansia*, *uncultured bacterium f Muribaculaceae*, *Allobaculum*, *Lachnospiraceae NK4A136 group*, *Rummeliibacillus* and *Parasutterella* significantly increased in the vitamin A supplementation groups. Interestingly, most of these genera have been found to interact with the host immune system. *Akkermansia*, which belongs to the phylum *Verrucomicrobia*, has the potential to induce regulatory immunity [78]. Otherwise, the genus *Akkermansia* has also been suggested to be a potential biomarker of a healthy gut status [79] and to play a key role in maintaining the barrier function of the digestive tract and controlling metabolism-induced inflammation and fat storage, using mucins as an energy source to stimulate goblet cells to produce mucus, and enhance mucus layer thickness and the intestinal barrier [67,80]. In the present study, we found that vitamin A supplementation significantly increased the abundance of *Lachnospiraceae NK4A136 group* and *Allobaculum*, which is consistent with the studies by Xiao et al. [74] and Lee and Ko [81]. *Lachnospiraceae NK4A136 group* has been reported to be a discriminative feature of gut dysbiosis [82]. *Allobaculum* was strongly inversely correlated with the expression of inflammation markers (*Saa3* and *Pai1*) [83]. *Parasutterella* may exert potential beneficial effects on intestinal mucosal homeostasis [84]. However, in contrast to our finding, vitamin A deficiency increased the abundance of *Parasutterella* in female mice [74]. This difference is likely attributed to the diet [85]. In this study, mink needed a large amount of fat in the diet to withstand cold weather in the winter [86], and diets containing a high content of animal proteins and saturated fats increased bile secretion, augmenting bile acids in the intestine [87]. In addition, the genus *Parasutterella* is reported to be associated with bile acid maintenance and cholesterol metabolism [84]. Therefore, the increase in *Parasutterella* may be due to the higher fat content in the diet, which will require an increased amount of bile acid secretion for digestion [88]. The *Muribaculaceae* family, previously named *S24–7* based on its predicted potential to degrade complex carbohydrates, was previously grouped into three trophic guilds with different degradation capacities: a-glucans, complex plant cell wall glycans (hemicellulose and pectin) and host-derived glycans [89,90]. The genus *Rummeliibacillus* belongs to the *Bacillaceae* family, which was shown to possess a variety of open reading frames predicted to be involved in glycogen synthesis and cellulose metabolism, as suggested by metagenome sequencing and analysis [91]. These results suggest that vitamin A deficiency leads to the destruction of the gastrointestinal mucosal barrier and increases the risk of mucosal infection.

We also applied PICRUSt to predict the potential functions, and the results show that glycan biosynthesis and metabolism, transport and catabolism pathways were significantly more abundant in the vitamin A supplementation groups than those in the CON group at KEGG level 2. Symbiotic microorganisms that reside in the intestine are adept at foraging glycans and polysaccharides, including those in dietary plants (starch, hemicellulose and pectin) and host mucus (O-linked glycans) [92]. *Muribaculaceae* and *Lachnospiraceae* are the identified mucosal sugar foragers that actively use all or most O-glycan mucosal sugars [93]. As discussed above, the results show that the relative abundances of *uncultured bacterium f Muribaculaceae* and *Rummeliibacillus* significantly increased in the vitamin A supplementation groups, and they are reportedly associated with carbohydrate degradation, glycogen synthesis and cellulose metabolism. Hence, the results show that vitamin A may have the potential to regulate carbohydrate metabolism by the microbiota resident in the jejunum of mink. However, the limitation of this study is the relatively small size, which may affect the significant difference among the three groups, and studies with a large sample size are needed to further reveal the effect of vitamin A in regulating gut microbiota, which will provide more evidence of the role of vitamin A in affecting the growth and health of mink.

## 5. Conclusions

In conclusion, the present study revealed that 20,000 IU/kg vitamin A supplementation could promote mink growth by modulating intestinal development, and improving antioxidant capacity and intestinal mucosal barrier functions. We observed that dietary vitamin A supplementation can modulate the mink gut microbiome and host physiology to a healthier phenotype by increasing the relative abundance of beneficial bacteria, such as *Akkermansia*, *Allobaculum* and *Lachnospiraceae NK4A136 group*. Moreover, vitamin A deficiency had a negative impact on mink growth by decreasing the relative abundance of beneficial bacteria. However, the underlying mechanisms of vitamin A in immunity still need further investigation.

## Figures and Tables

**Table 1:** Composition and nutrient levels of experimental diets, %.

Ingredients	Content	Nutrient Levels ^2^	Content
Extruded corn	36	Metabolizable energy (MJ/kg)	16.44
Soybean meal	1.5	Dry matter	96.13
Meat meal	7.8	Crude protein	35.36
Corn gluten meal	1.5	Ether extract	16.53
Chicken meal	14.0	Carbohydrates	34.10
Fish meal	25.5	Ash	10.14
Soybean oil	11.8	Ca	2.66
DL-Methionine	0.5	P	1.35
L-lysine HCL	0.4	Vitamin A IU/kg	1558
Premix ^1^	1.0		
Total	100.0		

^1^ Premix provided following per kilogram of diet: vitamin D_3_ 2200 IU; vitamin E 220 mg; vitamin K_3_ 1 mg; vitamin B_1_ 20 mg; vitamin B_2_ 10 mg; vitamin B_6_ 10 mg; vitamin B_12_ 0.1 mg; nicotinic acid 40 mg; pantothenate 22 mg; folic acid 1 mg; biotin 1 mg; choline chloride 400 mg; vitamin C 120 mg; Cu 20 mg; Fe 80 mg; Zn 40 mg; Mn 16 mg; I 0.5 mg; Se 0.12 mg; Co 0.2 mg. ^2^ ME and carbohydrates were calculated values, while the others were measured values.

**Table 2:** Effect of dietary vitamin A supplementation on the growth performance of growing-furring male mink.

Items	Treatments			SEM	*p*-Value
	CON	LVitA	HVitA		
Initial body weight, kg	1.98	1.97	1.98	0.035	0.98
Final body weight, kg	2.24	2.31	2.30	0.037	0.71
Average daily gain, g/d	5.74 ^a^	7.66 ^b^	7.01 ^ab^	0.33	0.048
Body length, cm	46.1	47.85	47.44	0.61	0.74

In the same row, values with no letter or the same letter superscripts mean no significant difference (*p* > 0.05), ^a,b^ while different small letter superscripts mean significant difference (*p* < 0.05). The same as below.

**Table 3:** Effect of vitamin A supplementation on jejunum morphology in fur-growing male mink.

Items	Treatments			SEM	*p*-Value
	CON	LVitA	HVitA		
villus height, μm	1115.12 ^a^	1423.62 ^c^	1252.79 ^b^	33.93	0.001
crypt depth, μm	567.55	576.71	539.86	11.22	0.484
intestinal wall thickness, μm	341.42	367.63	345.23	9.76	0.479
villus height/crypt depth	1.98 ^a^	2.49 ^b^	2.33 ^b^	0.84	0.001

In the same row, values with no letter or the same letter superscripts mean no significant difference (*p* > 0.05), ^a–c^ while different small letter superscripts mean significant difference (*p* < 0.05). The same as below.

## Data Availability

The datasets generated in this study can be found in online repositories. The sequencing datasets generated during this study are available at NCBI BioProject: PRJNA646038.
